# Identification of a Novel Nematotoxic Protein by Challenging the Model Mushroom *Coprinopsis cinerea* with a Fungivorous Nematode

**DOI:** 10.1534/g3.115.023069

**Published:** 2015-11-17

**Authors:** David Fernando Plaza, Stefanie Sofia Schmieder, Anna Lipzen, Erika Lindquist, Markus Künzler

**Affiliations:** * Institute of Microbiology, Department of Biology, ETH Zürich, 8093 Zürich, Switzerland; † Genomic Technologies, Joint Genome Institute, Walnut Creek, California 94598

**Keywords:** basidiomycete, fungal defense, RNA sequencing, CCTX2, transcriptomics

## Abstract

The dung of herbivores, the natural habitat of the model mushroom *Coprinopsis cinerea*, is a nutrient-rich but also very competitive environment for a saprophytic fungus. We showed previously that *C. cinerea* expresses constitutive, tissue-specific armories against antagonists such as animal predators and bacterial competitors. In order to dissect the inducible armories against such antagonists, we sequenced the poly(A)-positive transcriptome of *C. cinerea* vegetative mycelium upon challenge with fungivorous and bacterivorous nematodes, Gram-negative and Gram-positive bacteria and mechanical damage. As a response to the fungivorous nematode *Aphelenchus avenae*, *C. cinerea* was found to specifically induce the transcription of several genes encoding previously characterized nematotoxic lectins. In addition, a previously not characterized gene encoding a cytoplasmic protein with several predicted Ricin B-fold domains, was found to be strongly upregulated under this condition. Functional analysis of the recombinant protein revealed a high toxicity toward the bacterivorous nematode *Caenorhabditis elegans*. Challenge of the mycelium with *A. avenae* also lead to the induction of several genes encoding putative antibacterial proteins. Some of these genes were also induced upon challenge of the mycelium with the bacteria *Escherichia coli* and *Bacillus subtilis*. These results suggest that fungi have the ability to induce specific innate defense responses similar to plants and animals.

Interactions between organisms can be either beneficial or detrimental for the partners involved. In order to avoid the detrimental effects caused by antagonistic organisms, in particular multicellular organisms have evolved sophisticated defense strategies, comprising mechanisms to recognize the presence of other organisms, and to distinguish between self and nonself (Zhang *et al.* 2010; Zipfel 2008), as well as the production of defense molecules, such as proteins (Bleuler-Martinez *et al.* 2011; Gallo and Hooper 2012; Vandenborre *et al.* 2011), RNAs (Liu *et al.* 2012), peptides (Walton *et al.* 2010), and secondary metabolites (Engel *et al.* 2002; Rohlfs and Churchill 2011; Spiteller 2008). It has been hypothesized that such defense systems originally evolved to prevent the fusion of somatic conspecifics that were genetically different (Muller and Muller 2003; Srivastava *et al.* 2010). Cytoplasmic and transmembrane pattern recognition receptors (PRRs) specifically recognizing conserved microbe- (MAMPs) or damage- (DAMPs) associated molecular patterns have been described and characterized in many animals including cnidarians (Bosch 2013), annelids (Skanta *et al.* 2013), mollusks (Yoshino *et al.* 2008), arthropods (Wang and Ligoxygakis 2006), and chordates (Hopkins and Sriskandan 2005). Plants also recognize MAMPs and DAMPs using PRRs, and share several other innate defense mechanisms with animals, including the production of reactive oxygen (Gleason *et al.* 2011; Nathan and Cunningham-Bussel 2013; Liu *et al.* 2010) and nitrogen (Prior *et al.* 2009; Nurnberger *et al.* 2004) species as well as the biosynthesis of toxic proteins (Vandenborre *et al.* 2011), antimicrobial peptides (Benko-Iseppon *et al.* 2010; Tennessen 2005), and secondary metabolites (Bednarek 2012). The signaling pathways involved in animal and plant defense responses are conserved (Pedley and Martin 2005), and often lead to differential gene expression, suggesting that innate defense systems are an ancient and widespread trait that appeared very early in evolution. Accordingly, fungi are expected to also deploy innate defense mechanisms but, to date, not much is known about these mechanisms.

A main aspect of defense is the ability of an organism to distinguish between self and nonself. Fungi are known to distinguish between compatible or noncompatible cells of their own kind by their mating type system (Bidard *et al.* 2013; Hall *et al.* 2010) or by a mechanism referred to as vegetative heterokaryon incompatibility (HI) (Bidard *et al.* 2013; Hutchison *et al.* 2009). The latter mechanism has been well characterized in the filamentous ascomycetes *Podospora anserina* and *Neurospora crassa*, and involves the recognition of noncompatible hyphae via cytoplasmic proteins resembling PRRs, and containing HET and STAND domains, which leads to an extensive transcriptional response, including the induction of genes encoding toxins (Bidard *et al.* 2013; Hutchison *et al.* 2009). Little is known about the recognition of antagonists, including competitors, predators and parasites, by fungi, and the subsequent fungal responses affecting the interaction of the fungi with these organisms. In *Aspergillus nidulans*, it was shown that the master regulator of secondary metabolite production, LaeA, is responsible for the resistance of this fungus to predation by larvae of the fruit fly *Drosophila melanogaster* (Caballero Ortiz *et al.* 2013). In agreement with these results, challenge of the vegetative mycelium with fungivorous collembola induced the formation of fruiting bodies, and the synthesis of toxic secondary metabolites, suggesting that *A. nidulans* is able to respond to its predator by mounting an effective defense response (Caballero Ortiz *et al.* 2013; Doll *et al.* 2013). Similarly, *A. fumigatus* responded to the presence of actinomycetous bacteria by producing antibacterial polyketides (Schroeckh *et al.* 2009). This response of the fungus depended on direct physical interaction between the bacterial and fungal filaments and on the acetylation of histones (Nutzmann *et al.* 2011). Finally, analysis of the transcriptional response of the plant pathogenic fungus *Magnaporthe oryzae* to the bacterial antagonist *Lysobacter enzymogenes* allowed the identification of a class of potential antibacterial defense effector proteins (Mathioni *et al.* 2013).

We have recently shown that the coprophile model mushroom *Coprinopsis cinerea* transcribes a broad array of genes encoding putative defense proteins against bacterial competitors and animal predators constitutively in a tissue-specific manner (Plaza *et al.* 2014; Essig *et al.* 2014). In addition, the biosynthesis of two nematotoxic defense proteins, CGL1 and CGL2, was shown to be induced in the vegetative mycelium of *C. cinerea* upon challenge with the predatory nematode *Aphelenchus avenae* (Bleuler-Martinez *et al.* 2011). The specificity and the extent of this fungal defense response remained unclear, however. In order to resolve these issues, we assessed the transcriptional response of the vegetative mycelium of *C. cinerea* to nematode predation and bacterial coculture at a genome-wide level. The results of this study show that several loci encoding nematotoxic and potentially bactericidal proteins are specifically induced in response to nematode predation and bacterial cocultivation, respectively.

## Materials and Methods

### Strains and general cultivation conditions

*Escherichia coli* DH5α was used for cloning and plasmid amplification. *E. coli* strain BL21 (DE3) was used for protein expression and biotoxicity assays, and strain OP50 was used for maintenance of *Caenorhabditis elegans*. For these purposes, *E. coli* was cultivated on LB or NGM medium as described (Stiernagle 2006). *E. coli* strain Nissle 1917, and *Bacillus subtilis* strain 168 were used for challenging *C. cinerea* and cultivated as described below. *C. elegans* wild-type strain Bristol type (N2) was obtained from *Caenorhabditis* Genetics Center (CGC) and cultivated on NGM plates preseeded with *E. coli* OP50 as described (Stiernagle 2006). The fungivorous nematode *A. avenae* (a kind gift from Prof. Richard Sikora, University of Bonn, Germany) was propagated at 20° on vegetative mycelium of *Agaricus bisporus* pregrown on PDA plates (Difco). Vegetative mycelia of *C. cinerea* strain Okayama7 (O7; monokaryon) and A43mutB43mut (AmBm; homodikaryon), used for the challenge experiments and gene/cDNA cloning, respectively, were cultivated on YMG agar (0.4% yeast extract, 1% malt extract, 50 mM glucose, 1.5% agar) plates at 37°. Cultivation of primordia of strain AmBm was performed as described previously (Kues 2000).

### C. cinerea challenge experiment

*B. subtilis* 168 and *E. coli* Nissle 1917 were cultivated from frozen stocks for 18 hr at 37° on YMG plates. Single colonies were inoculated in 2.5 ml YMG broth (0.4% yeast extract, 1% malt extract, 50 mM glucose), and cultivated to stationary phase at 37° under constant shaking. Bacteria were then washed twice in sterile-filtered PBS (135 mM NaCl, 2.5 mM KCl, 10 mM Na_2_HPO_4_, and 17.5 mM KH_2_PO_4_; pH 7.4) and their OD_600_ was adjusted with PBS to 0.0005 before use. The fungal-feeding nematodes were cultivated as described above, and harvested by the Baermann funnel method (Staniland 1954). Bacterivorous *C. elegans* N2 was cultivated as described above and harvested by washing the plates with PBS. After harvesting, all nematodes were transferred to water agar plates supplemented with 200 µg/ml G418, 50 µg/ml Nystatin and 100 µg/ml Ampicillin, and incubated for 48 hr to eliminate all the residual bacteria or fungi. Before use, nematodes were suspended and washed twice in sterile-filtered PBS and adjusted to a density of 2500 worms/ml. Individual single colonies of *C. cinerea* O7 vegetative mycelium were cultivated on separate 30-ml YMG agar plates covered with sterile cellophane discs at 37° in the dark for 96 hr. The other organisms were applied in 200 µl sterile-filtered PBS to the individual mycelial colonies and incubated for 72 hr at 24°: ca. 500 mixed-stage *A. avenae*; ca. 500 mixed-stage *C. elegans*; OD_600_: 0.0005 *B. subtilis* 168, or OD_600_: 0.0005 *E. coli* Nissle 1917. To mimic the tissue damage inflicted on the hyphae by the fungivorous nematode, the *C. cinerea* O7 mycelial colony was cut with a sterile scalpel every 24 hr for 72 hr. As negative control, 200 µl sterile-filtered buffer (PBS) was applied to the mycelial colony. The individual challenge experiments were performed in triplicate. Immediately after the challenge, the individual mycelial colonies were harvested and flash frozen using liquid nitrogen in separate tubes and stored at –80°.

### Extraction of total RNA

The frozen mycelium was lyophilized in a VirTis Freezemobile for 18 hr. Lyophilized tissue (20 mg/replicate/treatment) was lysed in three FastPrep FP120 homogenization steps of 45 sec at 4.5, 5.5 and 6.5 m/sec in the presence of 250 mg 0.5-mm glass beads, while cooling the samples for 5 min on ice between the steps. RNA was extracted from the lysed tissue using 1 ml Qiazol (Qiagen), and 0.2 ml chloroform (ReagentPlus, Sigma-Aldrich). The mixture was centrifuged at 12,000 × *g* for 15 min at 4°, and RNA from the upper aqueous phase was washed incolumn using the RNeasy Lipid Tissue Mini Kit (Qiagen), and eluted in 60 µl RNase-free water. Concentration and quality of the purified RNA was determined with a Qubit (1.0) fluorometer (Life Technologies), and a Bioanalyzer 2100 (Agilent), respectively. Samples with a 260/280 nm ratio of 1.8–2.1, a 28S/18S ratio of 1.5–2 and no signs of rRNA degradation were used for library construction.

### Illumina HiSeq 2000 library construction and sequencing

A TruSeq Stranded mRNA Sample Prep Kit (Illumina) was used to construct expression libraries (three biological replicates per treatment). Briefly, total RNA (1 μg) from each biological replicate was poly(A)-enriched, and then reverse-transcribed into double-stranded cDNA in the presence of Actinomycin during first-strand synthesis. Double-stranded cDNA was fragmented, end-repaired, and adenylated before being ligated to TruSeq adapters and selectively enriched by PCR. Concentration and quality of the enriched libraries were assessed using Qubit (1.0) fluorometer and LabChip GX (PerkinElmer), showing an average fragment size of 260 bp. Libraries were normalized to 10 nM with 10 mM Tris-HCl pH 8.5 supplemented with 0.1% Tween 20. In addition, TruSeq PE Cluster Kit v3-cBot-HS (Illumina) was used to generate clusters from 10 pM pooled normalized libraries. Paired-end sequencing was performed on an Illumina HiSeq 2000 at 2 X 101 bp or single-end 100 bp using the TruSeq SBS Kit v3-HS (Illumina).

### Validation by qRT-PCR

RNA-seq results were validated by qRT-PCR. Single-stranded cDNA from three (*A. avenae* challenge or nonchallenge control) or two (scalpel-damaged mycelia) biological replicates was synthesized using Transcriptor Universal cDNA Master (Roche) from 2 µg total RNA. qRT-PCR reactions (20 µl) were mixed in three technical replicates per primer set and sample, containing 900 nM forward and reverse primers designed to span exon–exon junctions (Supporting Information, Table S1), 10 µl 2X FastStart Universal SYBR Green Master (Rox, Roche), and 1 ng/µl cDNA template. qRT-PCR was performed in a Rotor-Gene 3000 (Corbett Life Science) with the following thermal profile: a hold step at 95° for 15 min, followed by 40 cycles of 95° for 15 sec, 62° for 30 sec, and 72° for 30 sec. In order to control the specificity of amplification, the reaction was concluded with a melting curve analysis ramping from 55° to 99° in steps of 1° every 5 sec. PCR efficiencies and cycle thresholds were obtained using LinRegPCR 12, and differential expression ratios were calculated by the C_T_ difference formula (Schefe *et al.* 2006). Tubulin beta chain (CC1G_04743) was used as a housekeeping normalizer. In addition, water or 1 ng/µl RNA was included as a negative control reaction. To further validate the significance of the RNA-seq-derived differential expression analysis, the constitutive expression of an array of housekeeping loci commonly used in qRT-PCR normalization (Ferreira and Cronje 2012; Huggett *et al.* 2005; Langnaese *et al.* 2008; Silveira *et al.* 2009; Wan *et al.* 2011) was verified in the sequencing datasets after library size normalization.

### Bioinformatic analysis

To obtain gene counts, reads from each RNAseq library were first trimmed to a specific length to ensure that reads with a low quality 3′ end still aligned. To determine the trimming length, 50,000 reads were randomly selected from the sequence data set, then trimmed to varying lengths and aligned to the reference genome (Stajich *et al.* 2010). Next, mapping rates were compared between the different trim lengths. The trim length with the highest percentage of mapping was selected as the trim length for all reads from each library. Following trimming, all reads were aligned to the reference transcriptome using the Burrows-Wheeler Aligner (Li and Durbin 2009) (seed length = 25, maximum hits = 1). The genome of *C. cinerea* strain O7 that was used as a reference is available for download from http://genome.jgi.doe.gov/Copci1/Copci1.home.html (Coprinopsis_cinerea.transcripts.fasta). Using the alignment, the number of reads that aligned to each gene for each sample separately, were counted (in-house script), and used to produce the counts matrix. Only reads that aligned uniquely and on the reverse strand of the reference were counted. Illumina mapped reads were deposited in the ArrayExpress database (http://www.ebi.ac.uk/arrayexpress/) under the accession number E-MTAB-2763. To determine the percentage of loci showing baseline expression, five reads/locus were taken as a minimal threshold. Reads per kilobase of transcript per million mapped reads (RPKM) were calculated for every locus in order to scale-normalize all the samples according to library and transcript size. For this, the number of reads for a locus in a library was divided by the total size of the library in mega-reads (millions of reads of the library that were mapped to the reference genome) multiplied by the length of the transcript encoded by the locus in kilobases (thousands of bases). Fold change per locus and a Welch’s *t*-test comparing the different challenge and control libraries were computed using normalized sense-RPKMs (Table S2). One advantage of using a higher number of biological replicates per treatment than the two necessary to apply EdgeR’s exact test (Robinson *et al.* 2010), was the possibility of using Welch’s *t*-test for pairwise comparison. Volcano plots comparing every treatment with the negative control were constructed. A Welch’s *t*-test p-value ≤ 0.05 [–log10 (p-value) > 1.3] and fold change ≥ 4 [log2 (fold change) ≥ 2] were the criteria established to classify a locus as significantly induced in a treatment. Nonetheless, for comparison with Welch’s, we applied an EdgeR’s exact test to our dataset containing raw read-counts after estimating normalization factors (calcNormFactors), common dispersion (estimateCommonDisp), and tagwise dispersion (estimateTagwiseDisp). Thereafter, a table containing logFC, logCPM, p-value, and FDR for all the loci was retrieved from EdgeR (Robinson *et al.* 2010). The number of differentially expressed genes based on false discovery rate (FDR) ≤ 0.05, or the combination of FDR ≤ 0.05 and a logFC ≤ –2 or logFC ≥ 2 was estimated. When the number of genes showing significant differential expression derived from the three types of analyses [FDR ≤ 0.05 only, FDR ≤ 0.05 combined with logFC and Welch’s p-value ≤ 0.05 combined with log2 (fold change)] was compared, a combination of Welch’s p-value ≤ 0.05 and log2 (fold change) > 2 or < –2 provided the smallest number of differentially expressed genes, thus proving to be more stringent than the two estimations derived from the EdgeR analysis package (Table S3). These stringent differential expression criteria were used henceforth. Venn diagrams were constructed to identify commonly regulated loci among treatments (http://bioinformatics.psb.ugent.be/webtools/Venn/). Functional annotation based on sequence similarity for loci found to be significantly induced was deduced by PSI-BLAST (Altschul *et al.* 1997) and PHYRE2 using standard parameters (Kelley *et al.* 2015). SignalP 4.1 (Petersen *et al.* 2011) and TMHMM v. 2.0 (Sonnhammer *et al.* 1998) were used to predict the presence of secretion signal and transmembrane helices in differentially expressed loci.

### Cloning and heterologous expression of CCTX2

Total RNA extraction and cDNA synthesis from *C. cinerea* AmBm were performed as described previously (Plaza *et al.* 2014). In brief, cDNA was synthesized using cDNA transcriptor universal (Roche) from 2 µg of total RNA following the manufacturer’s instructions. The coding region of CCTX2 (CC1G_10077) was amplified from cDNA using primers 10077forNdeI (GGAGTCGGCATATGGCTCTCAACGAAGGTG) and 10077revNotI (GAATAGCGGCCGCCTACAACTCGGAGTGCTTG). The PCR product was cloned into the pET24b expression vector (Novagen) using the *Nde*I and *Not*I restriction sites. Cloning of CGL2 into pET24b was described previously (Walti *et al.* 2008). For protein expression, the plasmid was transformed into *E. coli* BL21 (DE3). Transformants were cultivated in LB medium containing 50 µg/ml kanamycin to an OD600 = 0.7 and protein expression was induced with 0.5 mM isopropyl-β-d-thiogalactopyranoside (IPTG) at 20° for 16 hr. Solubility of the protein was tested as previously described (Kunzler *et al.* 2010).

### Nematotoxicity assay

Nematotoxicity of CCTX2 was determined by feeding the recombinant *E. coli* expressing CCTX2 to *C. elegans* as described (Kunzler *et al.* 2010; Sabotic *et al.* 2012). Briefly, a liquid toxicity assay was performed using 20–30 synchronized L1 larvae. The larvae were incubated for 2 days at 20° with an *E. coli* BL21 culture at OD_600_ of 2, expressing CCTX2, the positive control CGL2 (Butschi *et al.* 2010) or harboring the ’empty’ vector plasmid pET24b. Toxicity was assessed by counting larvae developed into L4 stage or adulthood. The assay was done in quadruplicate. Welch’s *t*-test was performed to validate statistical significance of the differences observed.

### Protein expression analysis in C. cinerea

For protein expression analysis, vegetative mycelium of *C. cinerea* O7 was challenged with *A. avenae* and *C. elegans* as described above for RNA sequencing analysis. Whole cell protein extracts of the respective samples were prepared as described in (Bleuler-Martinez *et al.* 2011). Induction of CGL2 and CCTX2 in the mycelial samples was verified by immunoblotting using protein-specific polyclonal antisera (1:5000). The antiserum against CGL2 has been described previously (Boulianne *et al.* 2000). The antiserum against the purified recombinant CCTX2 was raised in rabbits by Seramun Diagnostica GmBH (Berlin, Germany). Expression of CCTX2 was performed as described above, and purification of the protein was achieved by affinity chromatography on a Sepharose column.

### Data availability

Reference for data available in public repository: Illumina mapped reads were deposited in the ArrayExpress database (http://www.ebi.ac.uk/arrayexpress/) under the accession number E-MTAB-2763.

## Results

### General features of the analyzed C. cinerea transcriptomes

Illumina RNA-seq stranded libraries were constructed and sequenced in biological triplicates for mycelial colonies of the monokaryotic *C. cinerea* strain Okayama7 (O7) challenged with bacterivorous (*C. elegans* N2) and fungivorous nematodes (*A. avenae*), gram-positive (*B. subtilis* 168), and gram-negative bacteria (*E. coli* Nissle 1917), mechanical damage or the application of buffer (negative control). Sequencing of these libraries showed that 82–84% of the suggested 13,393 open reading frames (ORF) in the genome of *C. cinerea* were significantly transcribed (more than five reads mapping to the ORF). A total 753 million sense and antisense reads (100 bases each) were mapped to the 37.5 Mb genome of *C. cinerea* O7, accounting for a sequencing output of nearly 75 billion bases (2000 times the genome size) (Table 1 and Table S2). Illumina mapped reads were deposited in the ArrayExpress database (http://www.ebi.ac.uk/arrayexpress/) under the accession number E-MTAB-2763.

**Table 1:** General features of *Coprinopsis cinerea* mycelia exposed to different biotic and abiotic stress conditions

	Mapped Reads (Sense + Antisense)							
Treatment	Replicate 1^a^	% of Total*^b^*	Replicate 2^a^	% of Total*^b^*	Replicate 3^a^	% of Total*^b^*	Total Mapped	Mean % of Loci Transcribed
O7 + PBS	3.31E+07	81.1	4.14E+07	78.6	4.54E+07	83.1	1.20E+08	82.3
O7 + *A. avenae*	2.51E+07	53.7	4.90E+07	82.6	3.87E+07	75.5	1.13E+08	82.1
O7 + *C*. *elegans*	3.14E+07	77.1	5.28E+07	78.9	4.31E+07	83.0	1.27E+08	82.4
O7 + mechanical damage	2.40E+07	80.5	5.14E+07	80.5	4.08E+07	82.8	1.16E+08	82.6
O7 + *E. coli*	5.53E+07	79.4	4.77E+07	79.5	5.09E+07	82.0	1.54E+08	82.5
O7 + *B. subtilis*	4.86E+07	81.0	3.78E+07	83.1	3.63E+07	83.0	1.23E+08	83.3
Total no. of mapped reads							7.53E+08*^c^*	
Approximate read length							100 bases	
Approximate total output							75 Gigabases	

a Biological replicates.

b Percentage of the total number of sequenced reads that was mapped to the reference genome.

c Total number of mapped reads: 7.53E+08; Approximate read length: 100 bases; Approximate total sequence output: 75 Gbases.

### Identification of a novel nematotoxic protein based on the analysis of the differential transcriptome of C. cinerea challenged with the fungivorous nematode A. avenae

**Figure 1:**
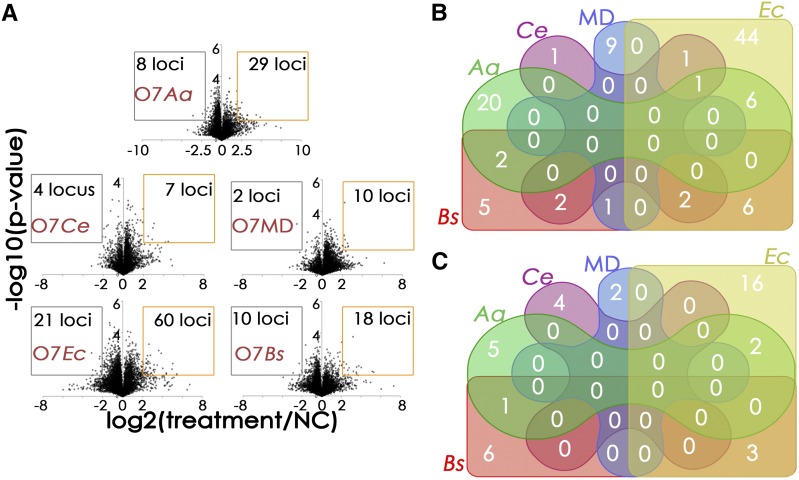
Challenge of *Coprinopsis cinerea* vegetative mycelium by *Aphelenchus avenae* predation and bacterial cocultivation induce treatment-specific defense responses at the transcriptional level. (A) Volcano plots showing the genome-wide differential expression analysis of *Coprinopsis cinerea* O7 challenged with the fungivorous nematode *A. avenae* (O7*Aa*), the bacterivorous nematode *Caenorhabditis elegans* N2 (O7*Ce*), *Escherichia coli* Nissle 1917 (O7*Ec*) and *Bacillus subtilis* 168 (O7*Bs*) relative to the application of buffer as negative control (NC). As additional treatment, supposedly mimicking the mechanical damage of hyphae inflicted by fungivore feeding, *Coprinopsis cinerea* vegetative mycelium was repeatedly cut with a scalpel (O7MD). Genes showing log2 (treatment/NC) ≥ 2 or ≤ –2, and –log10 (Welch's *t*-test-derived p-values calculated from three biological replicates) ≥ 1.3 were considered to be significantly upregulated (orange boxes) or downregulated (gray boxes). (B) Venn’s diagram computed for genes significantly upregulated by the fungivorous nematode *A. avenae* (*Aa*), the bacterivorous nematode *Caenorhabditis elegans* (*Ce*), the scalpel-inflicted hyphal damage (MD), *E. coli* (*Ec*) or *B. subtilis* (*Bs*). (C) Venn’s diagram computed for genes significantly downregulated by *A. avenae* (*Aa*), *Caenorhabditis elegans* (*Ce*), the scalpel-inflicted hyphal damage (MD), *E. coli* (*Ec*) or *B. subtilis* (*Bs*).

One of the goals of this study was the identification of novel defense effectors of *C. cinerea* against predatory nematodes based on the analysis of the set of *C. cinerea* genes upregulated upon challenge with these organisms. To achieve this goal, vegetative mycelium of *C. cinerea* strain O7 was challenged with the fungivorous nematode *A. avenae* for 72 hr, and the poly(A)-positive transcriptome of the challenged mycelial colonies was analyzed by RNA-seq and compared to the transcriptome of the buffer control. Genes displaying p-values ≤ 0.05 and expression fold changes (treatment/negative control or negative control/treatment) ≥ 4 were considered to be significantly upregulated or downregulated. This analysis led to the identification of eight *C. cinerea* genes being significantly downregulated and 29 genes being significantly upregulated upon challenge with *A. avenae* (Figure 1A, Table 2, and Table 3). Transcription of some of these genes responded exclusively to the presence of *A. avenae*, whereas the transcription of some was influenced also by the other biotic stresses applied in this study (Figure 1, B and C, Table 2, and Table 3) (see below).

**Table 2:** *Coprinopsis cinerea* Okayama7 protein-encoding genes significantly upregulated in response to the applied treatments

Treatment/Locus	PSI-Blast/PHYRE2 Prediction	SignalP	TMHMM	Pfam Cross-Reference	*Aa*/NC	*Ce*/NC	MD/NC	*Ec*/NC	*Bs*/NC
*A. avenae* (*Aa*)									
CC1G_01501	CBM-containing secreted protein	Y	0		4.1	—	—	—	—
CC1G_02104	Peroxidase	Y	0	PF11895; PF00141	5.4	—	—	—	—
CC1G_02355	Hypothetical secreted protein	Y	0		4.4	—	—	—	—
CC1G_02622	Ankyrin-repeat protein	N	0	PF12796	5.2	—	—	—	—
CC1G_03076	GH24 lysozyme	Y	0	PF00959	5.2	—	—	—	—
CC1G_04169	WSC domain-containing secreted protein	Y	0	PF00734; PF09362	5.2	—	—	—	—
CC1G_05003	CGL1 galectin	N	0	PF00337	5	—	—	—	—
CC1G_05005	CGL2 galectin	N	0	PF00337	4.1	—	—	—	—
CC1G_05809	Glycosyl hydrolase family 18 protein	Y	0		5.8	—	—	—	—
CC1G_06698	RTA1-like protein	N	7	PF04479	20.4	—	—	—	—
CC1G_08057	Wnt-like secreted protein	Y	0		4.4	—	—	—	—
CC1G_08593	Hypothetical secreted protein	Y	0		6.2	—	—	—	—
CC1G_09966	NmrA-family protein	N	0	PF05368	4.9	—	—	—	—
CC1G_10077	Ricin B-fold domain-containing protein	N	0	PF14200	4.9	—	—	—	—
CC1G_10726	Hypothetical secreted protein	Y	0		28.4	—	—	—	—
CC1G_11792	Hypothetical membrane protein	N	1		5	—	—	—	—
CC1G_11847	Defensin-related protein	Y	0		9.4	—	—	—	—
CC1G_12246	Hypothetical transmembrane protein	N	7		6.8	—	—	—	—
CC1G_14365	Hypothetical protein	N	0		6.1	—	—	—	—
CC1G_14558	Hypothetical protein	N	0		5.6	—	—	—	—
*C. elegans* (*Ce*)									
CC1G_04017	Hypothetical protein	N	0		—	5.8	—	—	—
Mechanical damage (MD)									
CC1G_02583	Membrane-anchored glycosylhydrolase	N	1		—	—	6	—	—
CC1G_03098	Ras-like GTPase	N	0	PF01926	—	—	4.8	—	—
CC1G_03514	Hypothetical protein	N	0		—	—	4.3	—	—
CC1G_09359	Hypothetical protein	N	0		—	—	4.9	—	—
CC1G_10157	Hypothetical protein	N	0		—	—	5	—	—
CC1G_11387	Cyclopropane-fatty-acyl-phospholipid synthase esynthase	N	0	PF02353	—	—	4.5	—	—
CC1G_11620	Hypothetical protein	N	0		—	—	4.6	—	—
CC1G_12367	Hypothetical transmembrane protein	N	7		—	—	5.7	—	—
CC1G_15356	Hypothetical protein	N	0		—	—	5.9	—	—
*E. coli* (*Ec*)									
CC1G_00122	Cytochrome P450	N	0	PF00067	—	—	—	4.1	—
CC1G_01525	Transaldolase	N	0	PF00923	—	—	—	4.5	—
CC1G_02052	YCII-related domain protein	N	0	PF03795	—	—	—	5.6	—
CC1G_02062	N-alpha-acetyltransferase 60-like	N	0	PF00583	—	—	—	9.4	—
CC1G_02166	Hypothetical membrane protein	N	1		—	—	—	9.3	—
CC1G_02345	Malate synthase	N	0	PF01274	—	—	—	5.7	—
CC1G_02382	Lipolytic enzyme	Y	0	PF00657	—	—	—	5.4	—
CC1G_02862	Snoal-like polyketide cyclase family protein	Y	0		—	—	—	6.5	—
CC1G_02908	Alcohol dehydrogenase	N	0	PF08240; PF00107	—	—	—	4.8	—
CC1G_03339	Fasciclin-domain containing secreted protein	Y	0	PF02469	—	—	—	4.8	—
CC1G_03442	Endonuclease/exonuclease/phosphatase	Y	0	PF03372	—	—	—	5.1	—
CC1G_03541	Hypothetical secreted protein	Y	0		—	—	—	16.7	—
CC1G_04927	Hypothetical protein	N	3		—	—	—	5.9	—
CC1G_05515	FAD-dependent oxidoreductase	Y	0	PF01266	—	—	—	5.5	—
CC1G_05607	Ig-domain containing secreted protein	Y	0		—	—	—	5.1	—
CC1G_05864	LolT-1-like PLP-dependent aminotransferase	N	0	PF00266	—	—	—	7.1	—
CC1G_05914	Ammonium transporter	N	11	PF00909	—	—	—	32	—
CC1G_06488	Urea transporter	N	15	PF00474	—	—	—	5.5	—
CC1G_06620	Isocitrate lyase	N	0	PF00463	—	—	—	4.5	—
CC1G_06972	NAD(P)H-dependent dehydrogenase	N	0	PF07992	—	—	—	4.1	—
CC1G_07061	Hypothetical protein	N	0		—	—	—	4.8	—
CC1G_07630	MAPEG protein	N	0	PF01124	—	—	—	4.1	—
CC1G_07735	Mitochondrial carrier/telomere-binding protein	N	0	PF00153; PF10451	—	—	—	5	—
CC1G_08094	High affinity methionine permease	N	12	PF13520	—	—	—	6	—
CC1G_08394	Hypothetical protein	N	0		—	—	—	8.5	—
CC1G_08758	Hypothetical secreted protein	Y	0		—	—	—	4.3	—
CC1G_08822	WSC domain-containing protein	Y	0	PF01822	—	—	—	4.1	—
CC1G_08888	Acyl-coA carboxylate coA-transferase	N	0	PF13336; PF02550	—	—	—	9.1	—
CC1G_11310	Pria protein	Y	0		—	—	—	5.6	—
CC1G_11374	PLAC8-domain-containing protein	N	0	PF04749	—	—	—	4.3	—
CC1G_11786	Hypothetical transmembrane protein	N	4		—	—	—	6.1	—
CC1G_12758	Acyl-coA N-acyltransferase	N	0	PF13302	—	—	—	20.3	—
CC1G_12964	Citrate synthase	N	0	PF00285	—	—	—	4.1	—
CC1G_12996	Ricin B-fold domain-containing protein	Y	0	PF00652	—	—	—	4.3	—
CC1G_13124	Ammonium transporter	N	9	PF00909	—	—	—	7.9	—
CC1G_13213	Glycerophosphoryl diester phosphodiesterase	Y	0	PF03009	—	—	—	12.2	—
CC1G_13246	Hypothetical transmembrane protein	N	5		—	—	—	6.2	—
CC1G_13826	Hypothetical transmembrane protein	N	4		—	—	—	7.4	—
CC1G_14125	Mitochondrial carrier	N	3	PF00153	—	—	—	5.1	—
CC1G_14829	Glutathione-S-transferase	N	0	PF00043; PF13417	—	—	—	8.4	—
CC1G_15187	Hypothetical protein	N	0		—	—	—	4	—
CC1G_15681	Hypothetical protein	N	0		—	—	—	8.3	—
CC1G_15703	Cytochrome p450	N	0	PF00067	—	—	—	4.7	—
CC1G_08300	Hydrophobin	N	1		—	—	—	10.5	—
*B. subtilis* (*Bs*)									
CC1G_03042	GH24 lysozyme	Y	0	PF00959	—	—	—	—	5.6
CC1G_05798	CBM-containing secreted protein	Y	0		—	—	—	—	4.2
CC1G_09605	Hypothetical protein	N	0		—	—	—	—	8.9
CC1G_10004	Hypothetical secreted protein	Y	0		—	—	—	—	5.1
CC1G_08311	Monocarboxylate permease	N	11	PF07690	—	—	—	—	4.7
*Ec*+*Bs*									
CC1G_00718	Hypothetical secreted protein	Y	1		—	—	—	6.6	6.4
CC1G_05600	Hypothetical secreted protein	Y	0		—	—	—	10.9	11.7
CC1G_08056	Wnt-like secreted protein	Y	0		—	—	—	20.6	15.9
CC1G_08433	Hypothetical protein	N	0		—	—	—	42.8	11.1
CC1G_09365	Triacylglycerol lipase	Y	0	PF01083	—	—	—	6.8	11.5
CC1G_14477	GH24 lysozyme	N	0	PF00959	—	—	—	6.9	6.2
*Aa*+*Ec*									
CC1G_02441	Hypothetical protein	Y	0		6.6	—	—	5.2	—
CC1G_04734	Med17 domain-containing protein	Y	0		4.5	—	—	5.7	—
CC1G_07582	Hypothetical secreted protein	Y	0		8.9	—	—	5.1	—
CC1G_09529	Hypothetical secreted protein	Y	0		5	—	—	5.7	—
CC1G_10384	O-Methylsterigmatocystin oxidoreductase	N	0	PF00067	4.6	—	—	6.7	—
CC1G_06684	LysM domain-containing protein	Y	0	PF01476	4.2	—	—	15.4	—
*Ce*+*Ec*									
CC1G_02581	Hypothetical membrane protein	Y	2		—	8	—	31.1	—
*Aa*+*Bs*									
CC1G_05472	Wnt-like secreted protein	Y	0		9	—	—	—	6.7
CC1G_03047	GH24 lysozyme	N	0	PF00959	7	—	—	—	4.8
*Ce*+*Bs*									
CC1G_08818	Hypothetical secreted protein	Y	0		—	17.7	—	—	38.1
CC1G_13803	Hypothetical protein	N	0		—	7.2	—	—	12.7
*Aa*+*Ce*+*Ec*									
CC1G_13818	MFS general substrate transporter	Y	9		9.1	5.4	—	16.1	—
MD+*Bs*									
CC1G_15139	Metalloprotease	Y	0	PF05572	—	—	4		13.4
*Ce*+*Ec*+*Bs*									
CC1G_01042	PAP2 superfamily protein	Y	0		—	21.7	—	42.9	35
CC1G_05219	KapM protein	Y	0		—	7.5	—	14.5	9.3

As thresholds of significant differential expression, fold (treatment/negative control) ≥ 4 and Welch’s *t*-test p-value ≤ 0.05 (from three biological replicates per treatment) were used. Presence (Y) or absence (N) of secretion signal was computed with SignalP 4.1. Number of transmembrane helices was predicted using TMHMM v. 2.0 in the proteins encoded by differentially expressed genes. Pfam cross-reference IDs are shown when available.

**Table 3:** *Coprinopsis cinerea* Okayama7 protein-encoding genes significantly downregulated in response to the applied treatments

Treatment/Locus	PSI-Blast/PHYRE2 Prediction	SignalP	TMHMM	Pfam Cross-Reference	NC/*Aa*	NC/*Ce*	NC/MD	NC/*Ec*	NC/*Bs*
*A. avenae* (*Aa*)									
CC1G_09526	Endoglucanase-4	Y	0	PF03443	4.1	—	—	—	—
CC1G_09644	Alpha-galactosidase	N	0		5.8	—	—	—	—
CC1G_13605	Hypothetical protein	N	0		20.5	—	—	—	—
CC1G_13985	Hypothetical protein	N	0		6.7	—	—	—	—
CC1G_14464	Reverse transcriptase/ribonuclease H	N	0		6.2	—	—	—	—
*C. elegans* (*Ce*)									
CC1G_02106	Hypothetical protein	Y	0		—	6.7	—	—	—
CC1G_09458	Extracellular tungstate binding	N	0		—	4.1	—	—	—
CC1G_13558	Hypothetical protein	Y	0		—	5.1	—	—	—
CC1G_15088	Hypothetical protein	N	0		—	5.4	—	—	—
Mechanical damage (MD)									
CC1G_08269	Hypothetical protein	Y	0	PF14273	—	—	4.6	—	—
CC1G_08983	Hypothetical protein	N	7		—	—	6.7	—	—
*E. Coli* (*ec*)									
CC1G_01035	Hypothetical protein	N	0		—	—	—	8.8	—
CC1G_01577	Glycosyl hydrolase family 62 protein	Y	0	PF00734; PF03664	—	—	—	6.7	—
CC1G_01879	RNA recognition motif 2 partial	N	0	PF04059	—	—	—	4.1	—
CC1G_02999	Hypothetical protein	N	0		—	—	—	4.2	—
CC1G_06017	Tyrosinase central domain-containing protein	Y	0	PF00264	—	—	—	5.3	—
CC1G_08259	Leucine-rich repeat domain protein	N	0	PF12937	—	—	—	16.5	—
CC1G_10148	Hypothetical protein	N	0		—	—	—	4.1	—
CC1G_10470	Tyrosinase central domain-containing protein	Y	0	PF00264	—	—	—	8	—
CC1G_10494	Hypothetical protein	N	5		—	—	—	4.7	—
CC1G_11188	ycaC protein	N	2	PF00857	—	—	—	8.1	—
CC1G_11580	Hypothetical protein	N	0		—	—	—	4.5	—
CC1G_12408	Hypothetical protein	N	0		—	—	—	4.2	—
CC1G_12509	Galactose mutarotase-like protein	N	0		—	—	—	4.4	—
CC1G_14013	Cytochrome P450	N	1	PF00067	—	—	—	4.5	—
CC1G_14014	O-Methylsterigmatocystin oxidoreductase	N	0	PF00067	—	—	—	4.6	—
CC1G_14164	AlphaN-acetylglucosamine transferase	N	1		—	—	—	4.8	—
*B. Subtilis* (*bs*)									
CC1G_01487	Hypothetical protein	N	0		—	—	—	—	6.9
CC1G_04915	Hypothetical protein	N	4		—	—	—	—	9
CC1G_05337	Hypothetical protein	N	7		—	—	—	—	6.4
CC1G_05968	Hypothetical protein	N	1		—	—	—	—	5.6
CC1G_08393	Hypothetical protein	Y	1		—	—	—	—	6.2
CC1G_14873	Hypothetical protein	N	0		—	—	—	—	4.4
*Ec*+*bs*									
CC1G_03158	Hypothetical protein	Y	3		—	—	—	5.9	7.4
CC1G_09799	AgaK1 protein kinase	N	0	PF00069	—	—	—	5.9	7.1
CC1G_10006	Tyrosinase	N	0	PF00264	—	—	—	4.3	4.1
*Aa*+*ec*									
CC1G_06907	WD40 domain-containing protein	N	1		5.8	—	—	6.8	—
CC1G_15644	Hypothetical protein	Y	0		10.2	—	—	13.6	—
*Aa+bs*									
CC1G_15616	Extracellular tungstate binding	N	0		4.9	—	—	—	6.2

As thresholds of significant differential expression, fold (negative control/treatment) ≥ 4 and Welch’s *t*-test p-value ≤ 0.05 (from three biological replicates per treatment) were used. Presence (Y) or absence (N) of secretion signal was computed with SignalP 4.1. Number of transmembrane helices was predicted using TMHMM v. 2.0 in the proteins encoded by differentially expressed genes. Pfam cross-reference IDs are shown when available.

In order to validate the RNA-seq data of the *A. avenae* challenge, the expression of a selection of genes showing statistically significant induction in response to *A. avenae* was confirmed by qRT-PCR (Figure S1) and immunoblotting (Figure S2). As an additional validation of the RNA-seq data, the expression of several reported housekeeping loci (Ferreira and Cronje 2012; Huggett *et al.* 2005; Langnaese *et al.* 2008; Silveira *et al.* 2009; Wan *et al.* 2011) was not changed by any of the treatments applied to *C. cinerea* (Table S4).

**Figure 2:**
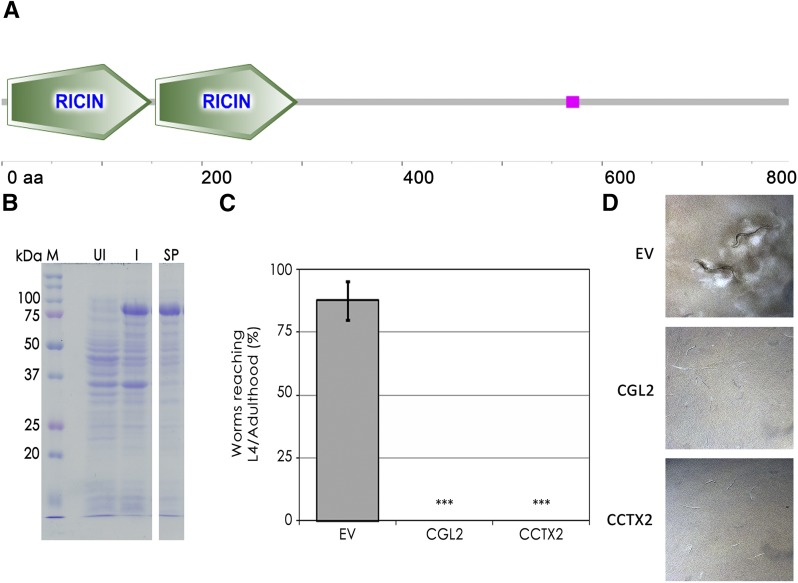
CC1G_10077-encoded CCTX2 is nematotoxic. (A) Schematic domain representation of CCTX2 predicting two Ricin-type beta-trefoil domains potentially involved in carbohydrate binding. (B) Expression of soluble CCTX2 in *E. coli*. The cDNA of CC1G_10077 was cloned, and CCTX2 was expressed in the cytoplasm of *E. coli* BL21(DE3). Extracts of uninduced (UI) and (I) cells, as well as soluble proteins from induced cells (SP), were run on SDS-PAGE and stained with Coomassie Brilliant Blue. The predicted molecular weight of CCTX2 is 89 kDa. (C) Nematotoxicity of CCTX2. CCTX2-expressing bacteria and control bacteria containing ’empty’ vector (EV), or expressing the previously characterized nematotoxic lectin CGL2, were fed for 48 hr to L1 larvae of *Caenorhabditis elegans* N2 in order to assess the toxicity of CCTX2 toward nematodes. A Welch’s *t*-test was computed to test the significance of the differences observed between treatments and the ’empty’ vector control. ***p-value ≤ 0.001. Bars represent the standard deviation calculated for four biological replicates. (D) Phase contrast micrographs of *Caenorhabditis elegans* N2 fed with *E. coli* BL21 containing ’empty’ vector (EV), or *E. coli* expressing CGL2 or CCTX2.

Among the set of genes that was specifically upregulated by *A. avenae* predation, CC1G_10726 and CC1G_06698 were the most highly induced, with fold changes of 20 and 28, respectively. Whereas the hypothetical protein encoded by CC1G_10726 did not, other than a consensus signal peptide for classical secretion (SignalP), show any sequence or structural similarity to any other functionally characterized protein in the currently available databases, CC1G_06698 codes for a seven-transmembrane domain protein that is homologous to the Rta1p protein of *Saccharomyces cerevisiae*. This protein may have a potential detoxifying function since it confers resistance of *S. cerevisiae* toward 7-aminocholesterol (Soustre *et al.* 1996). Consistent with previous results (Bleuler-Martinez *et al.* 2011), the two paralogous genes CC1G_05003 and CC1G_05005 coding for the nematotoxic galectins CGL1 and CGL2 (Butschi *et al.* 2010), respectively, were found to be specifically induced by *A. avenae* (Table 2 and Figure 1A). The set of *C. cinerea* genes specifically and significantly induced by *A. avenae* included the hitherto uncharacterized gene CC1G_10077 coding for a predicted cytoplasmic protein containing two putative RicinB-fold domains (Figure 1A, Figure 2A, and Table 2). In order to assess whether this protein, hereafter termed CCTX2 (*C.cinerea* toxin 2), was toxic to nematodes, the cDNA of CCTX2 was cloned and expressed in the cytoplasm of *E. coli* BL21. The heterologous expression of the cDNA yielded a soluble recombinant protein of the expected molecular weight of 89 kDa (Figure 2B). *C. elegans* L1 larvae were fed with *E. coli* expressing CCTX2, and the development of these larvae was compared to larvae fed with *E. coli* containing an ’empty’ vector control or expressing the previously characterized *C. cinerea* defense lectin CGL2 (Butschi *et al.* 2010). The protein CCTX2 was found to significantly impair the development of *C. elegans* larvae by arresting the worms at the L1 larval stage (Figure 2, C and D). These results suggest that CCTX2, together with the nematotoxic galectins CGL1 and CGL2, is expressed as a defense effector against nematodes feeding on *C. cinerea* vegetative mycelium.

### Considerable degree of specificity between differential transcriptomes of C. cinerea challenged with different types of abiotic and biotic stress

In order to evaluate the specificity of the transcriptional response of *C. cinerea* to the fungivorous nematode *A. avenae*, we determined the differential transcriptome of *C. cinerea* O7 vegetative mycelium upon challenge with the bacterivorous nematode *C. elegans* (Figure 1A, Table 2, and Table 3). In general, only a very low number of *C. cinerea* genes was upregulated or downregulated in response to *C. elegans* compared to the buffer control (Figure 1A). Consistent with our previous results (Bleuler-Martinez *et al.* 2011), this treatment did not induce the expression of any of the genes upregulated in response to *A. avenae*, suggesting that the mere presence of live nematodes is not sufficient to trigger the transcription of loci encoding defense effector proteins against this type of antagonist in *C. cinerea* (Figure 1B and Table 2). Fungivorous nematodes feed on fungal hyphae using a specialized feeding apparatus (stomatostylet), which resembles the needle of a syringe and allows the nematode to pierce the cell wall and membrane of fungal hyphae and suck out their contents (Ragsdale *et al.* 2008). In order to mimic this type of cell hyphal damage upon nematode predation, *C. cinerea* vegetative mycelium was repeatedly cut with a sterile scalpel (see *Materials and Methods*). The expression of nematotoxic lectins was not induced by this treatment (Figure 1B and Table 2), demonstrating that hyphal damage and resulting cytoplasmic leakage are not sufficient to trigger the nematotoxic response of *C. cinerea* to predation by *A. avenae*.

In order to compare the transcriptional responses of *C. cinerea* vegetative mycelium to animal predation and bacterial competition, the fungus was cocultivated with the Gram-positive bacterium *B. subtilis* 168 and the Gram-negative bacterium *E. coli* Nissle 1917. In the presence of *B. subtilis*, 28 genes of *C. cinerea* were found to be differentially expressed, with 18 genes being upregulated and 10 genes being downregulated (Figure 1A). Challenge of *C. cinerea* with *E. coli* resulted in a total of 81 differentially expressed genes including 60 significantly induced and 21 significantly repressed (Figure 1A). A comparison of the different sets of genes showing differential expression in response to the various types of biotic stress applied, revealed that the number of genes induced or repressed by more than one type of biotic stress was low, and that the transcriptional response of most genes was rather stress-specific (Figure 1, B and C, Table 2, and Table 3). Interestingly, the gene sets contain different members of the same gene family, which apparently differ in their responsiveness to biotic stress. One of these gene families encodes a set of homologous proteins containing a phage lysozyme domain of the glycosylhydrolase family 24 (GH24). Of this gene family, CC1G_03042 and CC1G_03076 were specifically induced by *B. subtilis* and *A. avenae*, respectively, whereas CC1G_03047 was upregulated in response to both *B. subtilis* and *A. avenae*, and CC1G_14477 was induced by both *B. subtilis* and *E. coli* (Table 2). Other examples of proteins that are encoded by paralogous genes and differed in the regulation of their biosynthesis in response to different types of biotic stress, are two secreted proteins containing a structurally predicted carbohydrate-binding module (CBM) (encoded by CC1G_01501 induced by *A. avenae* and CC1G_05798 induced by *B. subtilis*) and three secreted, cysteine-rich proteins with structural similarity to Wnt signaling proteins (Chu *et al.* 2013) (encoded by CC1G_08057 induced by *A. avenae*, CC1G_08056 induced by *E. coli* and *B. subtilis* and CC1G_05472 induced by *A. avenae* and *B. subtilis*) (Table 2).

## Discussion

Similar to plants (Spoel and Dong 2012), fungi are nonmotile organism lacking an adaptive immune system and specialized immune cells. Accordingly, both plants and fungi have evolved inducible innate defense systems that allow them to repel predators (Howe and Jander 2008; Caballero Ortiz *et al.* 2013; Bleuler-Martinez *et al.* 2011). In contrast to the well-characterized plant innate defense system, only scarce information about the fungal defense system is available.

Previous reports suggested that both filamentous asco- and basidio-mycetes are able to induce specific responses to biotic stresses (Bleuler-Martinez *et al.* 2011; Caballero Ortiz *et al.* 2013; Nutzmann *et al.* 2011). The specificity and the extent of this response at a genome-wide level was not investigated, however. Our results show that *C. cinerea* has the ability to discriminate between different abiotic (mechanical damage) and biotic stimuli, and respond to these stimuli by the induction or repression of specific gene sets. The increased production of cytoplasmic nematotoxic proteins (CGL1, CGL2, CCTX2) and secreted, potentially antibacterial proteins (family of putative phage lysozymes and/or defensin-related proteins) upon challenge of *C. cinerea* with the fungivorous nematode *A. avenae* and bacteria, respectively, suggests that fungi are able not only to distinguish between different antagonists but also to respond to these antagonists by inducing appropriate defense proteins.

It should be noted, however, that this general conclusion of our results is based on the postulated antibacterial activity of the upregulated family of phage lysozyme (GH24) domain-containing proteins, which still has to be demonstrated. Among the family members identified in this study, CC1G_03042 was specifically induced by the Gram-positive bacterium *B. subtilis*, while CC1G_14477 was upregulated in the presence of either *B. subtilis* or the Gram-negative bacterium *E. coli*. Surprisingly, two of the genes (CC1G_03076 and CC1G_03047) were also induced in mycelia challenged with *A. avenae*. As a possible explanation, this induction by a nematode may be due to contamination of *A. avenae* with bacteria. Arguments against this hypothesis are that the nematode was passaged over antibiotic-containing agar plates, and that challenge with *C. elegans*, which is raised on bacteria, did not lead to induction of these genes. Alternatively, the induction of antibacterial proteins by fungivorous nematodes may protect the hyphae from opportunistic bacteria feeding on the nutrient-rich cytoplasm leaking from the hyphae as a consequence of damage inflicted by nematode feeding.

Lysozymes are enzymes hydrolyzing the β-1,4-glycosidic bond linking monomers of N-acetylmuramic acid and N-acetylglucosamine in the bacterial peptidoglycan (Van Herreweghe and Michiels 2012). Lysozyme induction has been observed in coelomocytes from the earthworm *Eisenia andrei* exposed to *E. coli* (Joskova *et al.* 2009). Furthermore, the C-type lysozyme MgCLYZ from the mussel *Mytilus galloprovincialis* was shown to be have lytic activity against the Gram-negative bacteria *Vibrio anguillarum*, *Enterobacter cloacae*, *Pseudomonas putida*, *Proteus mirabilis* and *B. aquimaris* (Wang *et al.* 2013), indicating that some lysozymes are part of the innate defense response against Gram-negative microorganisms. Intriguingly, lysozymes of the GH24 family are only found in fungi, bacteria and bacteriophages, suggesting that the acquisition of these defense genes by fungi could be derived from a past horizontal gene transfer (HGT) event (Keeling and Palmer 2008). Accordingly, Metcalf *et al.* (2014) recently presented evidence for HGT of lysozymes of the GH25 family from bacteria to eukaryotes including fungi. A thorough biochemical analysis of these enzymes is needed to determine their role in fungal defense.

In contrast to the genes coding for potential antibacterial proteins, none of the genes coding for nematotoxic proteins was induced upon challenge of *C. cinerea* with bacteria. The specific induction of lectin-encoding genes CGL1 (CC1G_05003) and CGL2 (CC1G_05005) upon nematode predation in *C. cinerea* is a nice internal control of the results of this study since these lectins were previously shown to be toxic to the bacterivorous nematode *C. elegans* (Butschi *et al.* 2010), and to be induced upon challenge with *A. avenae* (Bleuler-Martinez *et al.* 2011). Interestingly, these genes are, in addition to upregulation upon nematode grazing, constitutively upregulated during fruiting body formation (Plaza *et al.* 2014; Boulianne *et al.* 2000). This dual regulation probably ensures constitutive protection of the reproductive organ and on-demand protection of the vegetative mycelium of the fungus.

One of the main results of this study is the identification of the novel nematotoxic protein CCTX2 based on its induction upon challenge with *A. avenae*. The protein contains several predicted Ricin B-fold domains shown to be present in lectins and protease inhibitors displaying nematotoxic or entomotoxic activity (Sabotic *et al.* 2012; Schubert *et al.* 2012). Accordingly, CCTX2 was shown to inhibit the development of *C. elegans* larvae, indicating that this protein acts as an effector in an inducible defense response against nematodes grazing on vegetative hyphae of *C. cinerea*. Induction of lectin- and toxin-encoding genes has also been observed during HI reactions between ascomycetes like *Podospora* and *Neurospora* (Bidard *et al.* 2013; Hutchison *et al.* 2009). The latter reactions were shown to be triggered by protein–protein interactions in the merged cytoplasm, and were proposed to have originally evolved as a recognition mechanism to detect pathogens (Paoletti and Saupe 2009). Despite the similarities with regard to target genes, no differential expression of *C. cinerea* HET and STAND domain-containing proteins was observed upon any of the treatments applied. These results suggest that HI and defense may use different regulatory pathways.

In conclusion, RNA-seq of *C. cinerea* challenged with fungivorous nematodes and bacteria showed that the induction of genes encoding nematotoxic lectins and potential antibacterial proteins is part of the transcriptional response of *C. cinerea* against predators and bacterial competitors. The specificity of this response suggests that *C. cinerea* has the ability to discriminate between different kinds of stimuli, and to regulate its gene expression accordingly. Furthermore, the Ricin B fold-containing protein CCTX2, induced by *A. avenae* challenge, was shown to be toxic to *C. elegans* larvae, demonstrating that this locus belongs to a transcriptional defense program triggered in *C. cinerea* upon nematode attack. This study supports previous reports (Bleuler-Martinez *et al.* 2011) suggesting the existence of elements (specific recognition and response) of inducible innate defense systems of plants and animals in *C. cinerea* and possibly multicellular fungi in general.

## 
